# LED-pumped room-temperature solid-state maser

**DOI:** 10.1038/s44172-025-00455-w

**Published:** 2025-07-09

**Authors:** Sophia Long, Lisa Lopez, Bethan Ford, François Balembois, Riccardo Montis, Wern Ng, Daan M. Arroo, Neil McN. Alford, Hamdi Torun, Juna Sathian

**Affiliations:** 1 Department of Mathematics, Physics and Electrical Engineering, Faculty of Engineering and Environment, Northumbria University, Newcastle upon Tyne, UK; 2 Université Paris-Saclay, Institut d’Optique Graduate School, CNRS, Laboratoire Charles Fabry, Palaiseau, France; 3 Department of Pure and Applied Sciences, University of Urbino, Campus Scientifico Enrico Mattei, Urbino, Italy; 4 Department of Materials, Imperial College London, London, UK

**Keywords:** Microwave photonics, Quantum optics, Inorganic LEDs

## Abstract

Room-temperature MASERs (Microwave Amplification by Stimulated Emission of Radiation) amplify electromagnetic waves at microwave frequencies with minimal noise. We demonstrate a cost-effective LED-pumped maser using pentacene-doped para-terphenyl as the gain medium. Here, we show that LED light, which is brightness-enhanced and guided via a cerium-doped yttrium aluminium garnet luminescent concentrator, achieves persistent maser emission at 1.45 GHz with a duration of 200 µs and a microwave output power of 0.014 mW, surpassing previous non-laser pumped systems. Operating at low voltage, the LED-pumped maser ensures safety, reduced costs, and simple integration. Potential applications include sensitive magnetic resonance imaging, portable atomic clocks, quantum technologies, and enhanced deep-space radio astronomy.

## Abstract (web summary)

Juna Sathian and colleagues demonstrate a room-temperature LED-pumped solid-state maser achieving persistent microwave emission. This advance opens opportunities for low-cost quantum technologies, deep-space communication, and sensing applications.

## Introduction

Masers can detect and amplify weak microwave signals while introducing minimal noise^1^. Although they have many potential applications, their usage has been limited to only a few specialised areas due to the requirement for vacuum, high magnetic fields, and extremely low temperatures^2,3^. The essential components of a maser include a gain medium, an excitation source (pump), and a resonant cavity. Recent advances have enabled room-temperature masers using organic crystalline materials, such as pentacene-doped para-terphenyl (PcPTP), coupled with cylindrical resonators and laser or lamp pumping^4–6^. Additionally, progress in miniaturised laser technology has facilitated the creation of compact laser-pumped masers^7^. Here, we report an alternative pumping system based on light-emitting diodes (LEDs), which offers several distinct advantages over traditional laser or lamp-based excitation sources. By directly coupling the LED output to the gain medium via a cerium-doped yttrium aluminium garnet (Ce:YAG) luminescent concentrator (LC), the design eliminates the need for complex optical alignment, enhancing simplicity and reproducibility. Moreover, the LED-based approach is safer, operating at low voltage and intensity, and is more cost-effective, utilising widely available components. Specifically, LED pumping offers an energy-efficient approach, minimises thermal loads in the gain medium compared to laser pumping, and ensures sufficient spectral overlap and photon density to drive quantum transitions in the pentacene-doped gain medium. By eliminating the need for high-power lasers and flashlamps, LED-pumped masers simplify both design and operation, broadening the practical applications of room-temperature maser technology. These features make LED-pumped masers particularly attractive for applications requiring scalability and cost-effectiveness. We address issues related to efficiency, thermal management, quantum mechanical considerations, and limitations of the existing pump systems.

The first solid-state maser that could operate at room-temperature was reported in 2012^4^, using photoexcited triplet-states in organic pentacene molecules housed in a microwave cavity containing a dielectric ring of sapphire and a pump laser. The pentacene molecules were stimulated at 585 nm using a dye laser (rhodamine 6G), generating pulses of 0.5 J at 1 ms pulse duration. This produced a burst of maser emission that lasted for 350 µs. The maser resulted in a peak microwave power output of 0.1 mW (−10 dBm), 100 × 10^6^ times higher than the hydrogen maser, with an optical pump threshold power of around 230 W. There have been subsequent reports of room-temperature masing using PcPTP^7–9^ and similar media^10^, pumped by a pulsed optical parametric oscillator in the range 570–620 nm, itself pumped by a frequency doubled pulsed neodymium-doped yttrium aluminium garnet (Nd:YAG) laser. A room-temperature maser was also demonstrated using an ensemble of nitrogen-vacancy centres in diamond^11^. In that case, the pump was a frequency-doubled Nd:YAG laser operating in continuous wave at 532 nm. In all these examples, the excitation source typically takes the form of a high-power laser due to the relatively high pump power thresholds.

In 2015, it was shown that using a strontium titanate (SrTiO_3_) dielectric resonator significantly reduced the optical pumping requirement by decreasing the magnetic mode volume by two orders of magnitude compared to the sapphire resonator used in the first reports of room-temperature masers. This led to a miniaturised maser that can be optically pumped by a non-laser source with lower power density, namely a xenon flash lamp at a useful peak optical power of 70 W^6^. A powerful burst of microwave energy was detected ~20 µs after the optical pulse began, reaching a peak power of 6 µW (−22.2 dBm). In 2020, a maser, pumped with a xenon flash lamp-luminescent concentrator was demonstrated with an output power of 3.1 µW (−25 dBm) for a peak pump power of 170 W^12^. While flashlamps can generate enough power to pump masers, they suffer from relatively short operating lifetimes of a few hundred to a few thousand hours. Flash lamp-pumped systems also exhibit high thermal loading because of the heat produced by the high-voltage gas discharge itself, extraneous absorption of the broadband emission by the host material, and other absorption transitions that do not contribute to the masing action^6,12^. Over the past 10 years, significant advancements in room-temperature optically pumped masers have established this field as an active and promising area of research. However, the pump sources remain a barrier to entering application fields and price-sensitive markets. The pump source properties are crucial, such as long lifetime, low cost, and specific optical properties, including a spectral match to the gain medium’s absorption (green-yellow band, 500–600 nm), high luminance, and a power density of ~100 Wcm^−2^. These characteristics are essential to achieve population inversion and sustained maser operation.

Driven by the lighting market, visible LEDs have been making steady progress for 25 years. Now, LEDs feature an even longer operating lifetime than laser diodes (>50,000 hrs) and a drastically lower cost. Considering LED costs are below 0.5 $/W and continuously decreasing^13^, LED pumping of masers appears very attractive from a cost perspective. Given recent investigations of LED-based systems for laser pumping^14–16^, the potential for LED-driven maser excitation has emerged as a compelling avenue for exploration^17^. Unfortunately, the pump wavelength range corresponds to the ‘yellow gap’^18^, where semiconductors are less efficient than in the blue or near-infra-red preventing pumping masers directly with LEDs. Indirect LED pumping via luminescent concentrators is another option. Indeed, Ce:YAG LCs pumped by blue LEDs are bright and powerful sources in the yellow-orange wavelength range^19–22^ and can produce quasi-continuous waves with peak irradiance reaching 10 kWcm^−2^ ^23^. Compared to other light sources, LED-pumped luminescent concentrators (LED-LC) have a significant advantage in energy scaling, which is directly related to the scaling of the dimensions of the luminescent crystal itself to add more pump LEDs. This design enables the system to operate collectively, distributing energy generation across multiple LEDs, minimising localised stresses, and supporting operation at a massive scale. This approach ensures a long lifetime, stability, and robustness, making it a reliable and efficient pumping method for masers. This pumping system has been successfully implemented for laser pumping of transition metal lasers^23–26^. Since the Ce:YAG emission matches the absorption band of pentacene molecules, this work aims to investigate the indirect LED pumping of a PcPTP maser using a Ce:YAG LC.

## Results

**Fig. 1:**
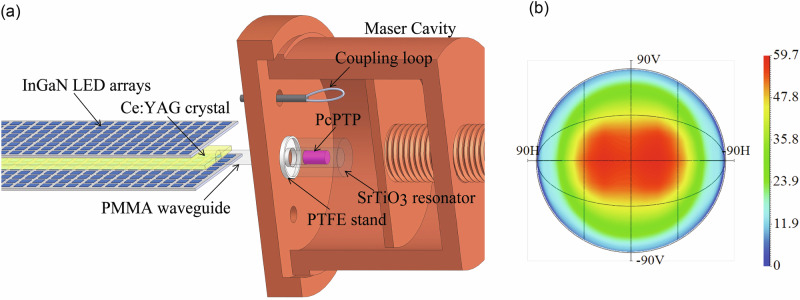
LED-pumped maser experimental setup and simulation of pump intensity profile. **a** InGaN LED-pumped maser experimental setup where the Ce:YAG luminescent crystal is used to effectively guide and concentrate the light into the PcPTP using a PMMA waveguide. A coupling loop connected to a coaxial cable, interfaces with the TE_01δ_ mode of the SrTiO_3_ resonator in the copper cavity, serving as an output coupler to extract the maser signal for subsequent measurement. **b** LightTools ray-tracing simulation shows the intensity distribution of the input pump profile at the PcPTP crystal. The figure shows the symmetric pump profile intensity mapped in azimuth and elevation, with colour-coded levels indicating relative luminance. The labels 90 V and 90H correspond to 90 degrees vertically and horizontally from the central peak intensity.

The LED pump system (Effilux) in this work uses 2120 blue Indium gallium nitride (InGaN) LEDs with an emission spectrum centred at 450 nm. The LEDs pump the LCs composed of two Ce:YAG parallelepipeds of 100 mm × 14 mm × 1 mm bonded with UV-cure adhesive NOA81. Ce^3+^ ions absorb the 450 nm light from the LEDs and emit it at longer wavelengths within the concentrator. The concentrator emits light between 530 nm and 650 nm, which is then directed to the concentrator’s edge (1 mm × 14 mm) through total internal reflection. To couple the light efficiently to the PcPTP crystal, we used a coupling waveguide, a 3 mm (diameter) × 10 mm (length) polymethyl methacrylate (PMMA) rod, as shown in Fig. 1a. The Ce:YAG LED pump system supports pulse durations that are adjustable between 7 µs and 200 µs. In the entire set of experiments, the repetition rate is fixed at 1 Hz. This choice is not due to inherent limitations but was made to ensure negligible thermal loads even at the highest duty cycle. At the output of the PMMA waveguide, we measure an energy of 15 mJ (with a Thorlabs pyroelectric energy sensor, ESC220C) for a duration of 200 µs, corresponding to a peak power of 75 W.

In this work, we used a 3 mm (diameter) × 6 mm (length) cylindrical PcPTP crystal enclosed in a SrTiO_3_ resonator (Fig. 1a). The 3 mm × 6 mm crystal was selected to optimise coupling with the TE_01δ_ mode of the SrTiO_3_ resonator and to balance the optical absorption required for population inversion with minimal attenuation. The LED-pumped maser is designed to optimise the launched pump power into the PcPTP. For this purpose, we chose a longitudinal pumping configuration, as shown in Fig. 1a. The SrTiO_3_ and PcPTP are placed on a polytetrafluoroethylene (PTFE) stand with a height of 2 mm. The PcPTP is attached to the PMMA waveguide by an index-matching adhesive (NOA81) with a refractive index of ~1.5. This index matching enables more efficient coupling of light into the PcPTP by reducing total internal reflections on the output face of the PMMA waveguide. By Monte Carlo ray-tracing simulation (LightTools), we estimated that the launched peak pump power reaches 130 W in the PcPTP during a 200 µs optical pulse. The absorption of optical pump light (~530–600 nm) by the pentacene molecules doped in the para-terphenyl crystal (Fig. 1a) was also estimated by LightTools to be 80% (Fig. 1b).

**Fig. 2:**
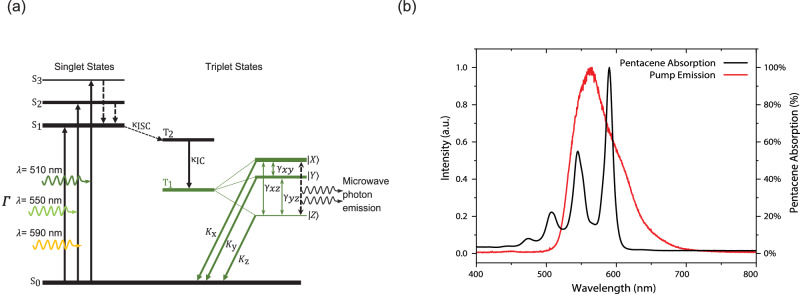
Spin dynamics and spectral overlap in PcPTP. **a** The Jablonski diagram, illustrating the state cycle that produces population-inverted spin-polarised triplet-state sublevels in PcPTP at zero magnetic field. Due to stimulated emission in the maser cavity, amplification of microwave photons occurs at 1.4493 GHz between $|X\rangle$ and $|Z\rangle$, triplet sublevels. The thickness of singlet and triplet states (black horizontal lines) denotes each level’s population, *Γ*: luminescent concentrator pump rate, ${K}_{{ISC}}$: intersystem crossing rate, ${K}_{{IC}}$: Internal conversion rate, ${\gamma }_{{xz}}$: spin-lattice relaxation rate between ${T}_{1}$ sublevels $|X\rangle$ and $|Z\rangle$, similarly for ${\gamma }_{{xy}}$ and ${\gamma }_{{yz}}$, ${K}_{X}$: decay rate from ${T}_{1}$ sublevels back down to ground state, similarly for ${K}_{y}$ and ${K}_{z}$ and completing a masing cycle. **b** The absorption spectrum of the pentacene and the emission of the Ce:YAG pump, showing the spectral overlap.

The active medium in this maser, PcPTP, exhibits strong spin properties due to its unpaired electrons positioned in the resonant cavity that traps and amplifies emitted microwave photons. Pentacene molecules consist of five condensed benzene rings arranged linearly. They possess two unpaired electrons in their highest occupied molecular orbital, leading them to be paramagnetic. When microwave fields interact with the unpaired electron spins in pentacene, molecules’ energy levels split. As a result, the spins begin to undergo coherent Rabi oscillations, causing the pentacene molecules’ population to periodically oscillate between different spin states. These oscillations ultimately lead to the establishment of population inversion, where the electrons become excited from their normal ground state to a longer-lasting metastable triplet state through intersystem crossing. At zero magnetic field, electronic interactions within the molecule cause the triplet sub-level energies to become non-degenerate, resulting in three unique sub-levels $|X\rangle$, $|Y\rangle$ and $|Z\rangle$ (Fig. 2a). These sub-levels are populated in a ratio of 9.5:2:1, with $|X\rangle$, having the highest energy and $|Z\rangle$ having the lowest^4,5^. The maser uses population inversion between the $|X\rangle$, and $|Z\rangle$ triplet-state sub-levels with an energy difference of ~1.45 GHz. When PcPTP is exposed to the input pump where there is a good spectral overlap (Fig. 2b), it moves from its ground state (S_0_) to its first excited singlet state (S_1_). This process triggers intersystem crossing, which causes an inverted spin population between the highest ($|X\rangle$) and lowest ($|Z\rangle$) spin sublevels of pentacene’s triplet ground state. Due to the high zero-field splitting, it is possible to use a microwave resonator without applying a d.c. magnetic field, making the experimental setup much simpler.

**Fig. 3:**
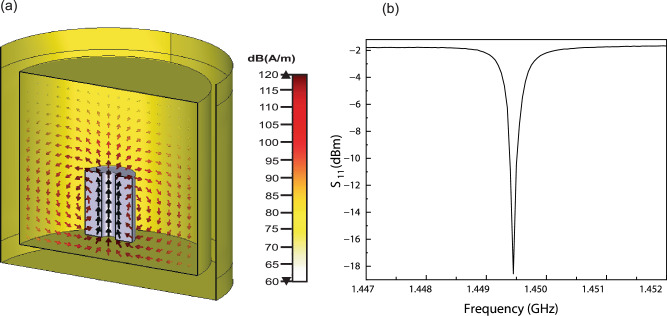
Maser cavity design and resonance characterisation. **a** Simulation image of SrTiO_3_ (light purple) inside the maser cavity (yellow). Plot of magnetic field density with directional arrows (red), implemented with electric wall boundary condition, setting tangential electric field components to zero and incorporating a boundary gap to minimise back reflections. SrTiO_3_ z-axis cutting plane displaying high concentration across the location of the PcPTP crystal gain medium. **b** Measured reflection coefficient (S_11_) using the cavity coupling loop exhibiting a resonant dip at 1.4493 GHz.

**Fig. 4:**
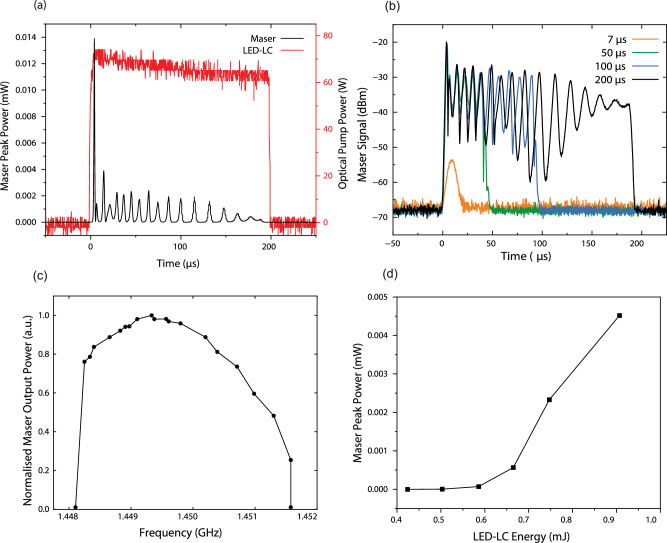
Temporal and spectral characteristics of maser output. **a** Temporal response of maser output power with a fixed LED pump duration of 200 µs (corresponding to a peak optical power of 75 W at the output of the PMMA waveguide), demonstrating immediate onset of maser oscillation. The maser exhibits persistent emission throughout the LED pump duration, with power variations attributable to Rabi oscillations. **b** Maser signal at different LED input pulse durations, exhibiting Rabi oscillations due to the coherent interaction between the microwave field and the pentacene triplet states. The oscillatory nature of the signal arises from population inversion dynamics, with variations in maser output power linked to pump duration and thermal effects. Compared to (**a**), the slightly reduced maser output at 200 µs results from thermal variations induced by the gradual buildup of pump energy over time. **c** Output power of the LED-maser at various resonant frequencies, with the highest power observed at 1.4493 GHz. **d** Maser peak output power as a function of LED-LC pump energy, showing threshold behaviour. The data is limited to the low-energy regime (up to 1 mJ) to highlight the onset of masing. Full-range output dynamics are presented in (**b**).

The maser cavity was designed using a commercially available finite-difference time-domain (FDTD) simulator (CST Studio Suite®). It is intended to maintain stationary waves at the designated microwave frequency and facilitate the TE_01δ_ mode, which has a high magnetic Purcell factor (Fig. 3a). The copper cavity was tuned to a resonant frequency of 1.4493 GHz using N9913A FieldFox Handheld RF Analyzer (4 GHz), (Fig. 3b). This was achieved by optimising the resonant mode and frequency of the cavity through precise adjustments of its dimensions and materials. The tuning process is crucial for enhancing the interaction between the cavity and the active medium, PcPTP, which has strong spin properties that lead to population inversion and Rabi oscillations. In this context, the reflection coefficient (S_11_) is a critical indicator of effective tuning, ensuring that most of the microwave energy is coupled into the cavity at the resonant frequency. This is essential for the efficient operation and overall performance of the maser. We observe persistent maser emission at 1.4493 GHz and Rabi flops using LED-LC pumping with a peak maser output power of 0.014 mW (−18.56 dBm). The temporal profiles (pump and maser) are plotted in Fig. 4a.

## Discussion

This work presents the first demonstration of a maser system using an LED pump, focusing on establishing the feasibility of maser operation with this approach. One of the advantages of LED pumping is the ability to tune the pump pulse duration easily. As shown in Fig. 4b, the temporal profile for pump duration ranges from 7 μs to 200 μs, representing the lower and upper bounds explored in our experiments. Intermediate durations, such as 50 μs and 100 μs, were also measured and aligned with the trends observed at these boundaries. The maser pulse duration is directly influenced by the input pulse duration, with shorter pulses leading to weaker, shorter-lived maser oscillations and longer pulses sustaining stronger and more defined oscillations due to extended population inversion. This behaviour is consistent with the theoretical framework of Rabi oscillations, which describes the coherent interaction between the microwave cavity field and the spin ensemble in the gain medium. The maser output begins a few microseconds after the onset of the LED pump and persists throughout the pump duration. This well-synchronised response confirms the temporal control of the masing process by the LED pump. The ability of the LED pulse to precisely dictate the maser emission profile provides strong evidence of the robustness and reproducibility of the LED-pumped maser system. It is important to note that the current LED power supply limits the pump pulse duration to 200 μs. However, this limitation can be addressed by upgrading to a more advanced LED module, which could enable longer maser pulses or even quasi-continuous-wave (quasi-CW) operation. Fully CW operation, while technically feasible, would require addressing additional challenges such as thermal management, LED power density, and material durability under sustained operation. Future developments in our laboratory aim to address these challenges, advancing maser design toward compact, energy-efficient, and long-duration operation. Our LED power supply is optimised for a fixed current (3.2 A per LED) corresponding to a peak pump power of *P*_*p*_ = 130 W launched into the pentacene. This means that we cannot measure the evolution of the maser output power as a function of the pump power and, thus, the pump power at the threshold. However, we can vary the upper state level population by reducing the pump duration below the upper state level lifetime (*τ*). This allows us to reach the oscillation threshold for a pump duration of *τ*_*p*_ = 7 µs. Using this approach, we estimate the equivalent steady-state pump power at threshold (*P*_*pth eq*_) using the relationship:

1 $${P}_{{pth\; eq}}={P}_{p}\left[1-{e}^{-\left(\frac{{\tau }_{p}}{\tau }\right)}\right]$$

For *τ*_*p*_ = 7 µs and *τ* = 22 µs, this corresponds to a launched pump power ${P}_{{pth\; eq}}$= 35 W. At maximum pump power, the pumping rate reaches a value of 3.7, meaning that the maser is far above the oscillation threshold.

The output power of the LED-pumped maser was assessed at various resonant frequencies, with the highest power detected at 1.4493 GHz (Fig. 4c). The TE_01δ_ mode was verified through simulations and experimental measurements. Finite-element simulations predicted the resonant frequencies and field distributions of the TE_01δ_ mode, while the cavity resonance was confirmed experimentally using a vector network analyser (VNA), N9917A FieldFox Handheld Microwave Analyzer (18 GHz). The resonator was carefully tuned to output power corresponding to the targeted TE_01δ_ mode. The maser output power, defined as the peak instantaneous power during the pulse, was measured using an MSO-X-3054A oscilloscope (500 MHz bandwidth) and an AD8318 logarithmic detector (1 MHz–8 GHz, 70 dB dynamic range). The logarithmic detector provided a voltage output proportional to the logarithm of the maser’s input power, which was converted to dBm and subsequently to milliwatts (mW) using calibrated parameters. Calibration was performed by validating the detector’s response against a signal generator with known input power and factoring in system gain. The optical pump power was recorded using a Thorlabs PDA100A2 amplified photodetector (320–1100 nm wavelength range) with adjustable gain, with its output voltage captured by the oscilloscope. This setup enabled accurate temporal measurements of the maser output and the optical pump power dynamics. For instance, the maser output signal measured −18.56 dBm at the resonant frequency, corresponding to 0.014 mW (14 µW). These details ensure the accuracy and reliability of the measurements, providing a clear methodology for assessing the maser’s performance. The graph in Fig. 4c illustrates a smooth tuning characteristic of the maser, with the output power reaching its maximum at a resonator frequency of 1.4493 GHz. The experiment involved manual adjustment of the resonant frequencies of the maser cavity by physically manipulating the plunger on top of the cavity, thereby altering its dimensions and subsequently modifying the frequency. The frequency variation was quantified using VNA, with the resultant peak maser output power observed within the frequency range of 1.4482–1.4509 GHz. This tuning range is primarily limited by the physical dimensions and dielectric properties of the SrTiO_3_ resonator, which determine the resonant frequency of the TE_01δ_ mode, and the fixed zero-field splitting of 1.45 GHz in the PcPTP gain medium. The coupling loop is also optimised for efficient power extraction at the operation band.

In conclusion, we have presented a type of solid-state maser that utilises LEDs and luminescent concentrators. The maser uses PcPTP as the gain medium and functions at a frequency of ~1.45 GHz. To our knowledge, this instance represents a unique case where a maser is indirectly pumped by LED. The microwave output power of the maser can attain a maximum of 0.014 mW, equivalent to −18.56 dBm. The output power achieved with the LED-pumped Ce:YAG system surpasses that of the flashlamp-pumped system by ~2.3 times for a transverse pumping configuration^6^ and 4.4 for a longitudinal pumping configuration^12^. The reasons for this are related to the LED-pumped luminescent concentrator ensuring both high power (two times higher than in ref. ^6^) and high pumping rate (nearly two times higher than in ref. ^12^) via the concentration effect in Ce:YAG. It should be noted that the LED-pumped Ce:YAG system used in this work is far from optimum. Indeed, only a part of the output power is coupled in the PcPTP because of dimension mismatch (14 mm for Ce:YAG versus 3 mm diameter for PcPTP). The output light that is not coupled into the waveguide could be recycled by additional mirrors on the output face, as in ref. ^27^.

The LED-pumped maser demonstrates significant potential to improve radiofrequency amplifiers and oscillator chains by offering a pathway toward more compact, durable, and affordable systems. This work highlights the inherent advantages of LED technology, including its scalability, low cost, and energy efficiency, which provide a foundation for advancing maser performance. Additionally, the long operational lifetime and precise control offered by LEDs could enable future improvements in energy efficiency, performance stability, and cost-effectiveness. With further development, LED-pumped masers could support a wide range of applications, such as enhancing magnetic resonance body scanners, advancing quantum optical coherence tomography, developing components for quantum computers, creating portable atomic clocks, and improving radio astronomy devices for deep space exploration.

## Methods

### Pentacene:p-terphenyl crystal growth

To prepare the maser gain medium for the described experiment, commercially sourced pentacene (TCI Europe NV) and p-terphenyl (Alfa Aesar, 99%) were subjected to further purification through sublimation and zone refining, respectively. These purified compounds were mixed in a mortar by liquid-assisted grinding (LAG) in the 0.1% (mol/mol) ratio, using toluene. The resulting lilac powder was dried at low pressure and sealed under an inert atmosphere in an 8 mm (inner diameter) boro-silicate tube, preliminarily heated under vacuum to remove humidity. The sample was crystallised from the melt using a home-built vertical tube Bridgman–Stockbarger furnace (*T* = 220 °C, speed = 2 mm/h), resulting in the pink crystalline sample.

### Maser pumping head

The LED pump system in this work uses 2120 blue Indium gallium nitride (InGaN) LEDs with an emission spectrum centred at 450 nm. When supplied with a peak current of 3.2 A, each LED produces a power of 2.5 W over an emitting area of 1 mm × 1 mm. The printed circuit boards of the LEDs are attached to a heatsink that is cooled by water, which is kept at 20 °C. The LEDs are used to pump the luminescent concentrator composed of two Ce:YAG parallelepipeds of 100 mm × 14 mm × 1 mm bonded together by UV curing (UV-cure adhesive NOA81, the refractive index of the cured polymer is 1.56). Ce^3+^ ions absorb the 450 nm light from the LEDs and are re-emitted within the concentrator. The concentrator emits light between 530 nm and 650 nm, which is then directed to the concentrator’s edge (1 mm × 14 mm) through total internal reflections. At the output of the Ce:YAG (surface 1 mm × 14 mm), we measure a power of 265 W, in air using Thorlabs pyroelectric energy sensor, ESC220C. To efficiently couple the light to the pentacene crystal, we use a PMMA coupling waveguide (3 mm diameter, 10 mm length) glued onto the Ce:YAG using UV-cure adhesive (NOA81). We measure an output power of 75 W at the output of the waveguide in air. The waveguide and the pentacene are then contacted by adhesive (NOA81) to increase the power transmitted to the pentacene. By Monte Carlo ray tracing simulations (LightTools), we estimated that the launched power reaches 130 W instead of 75 W in air. The absorption was also calculated by LightTools to be 80% at ~530–600 nm (Fig. 1b).

### Maser resonator design

The maser cavity was designed using a commercially available FDTD simulator (CST Studio Suite®). The dimension of the SrTiO_3_ cavity is 10.43 mm outer diameter (OD), 3 mm inner diameter (ID) and 14.82 mm in height (h). The cylindrical copper cavity 40 mm (ID) × 35 mm (h) enclosed the cylindrical PcPTP crystal of 6 mm × 3 mm and included a microwave port (a loop at the end of a coaxial cable) to extract microwave power produced by stimulated emission. The SrTiO_3_ and PcPTP are positioned on a PTFE stand, elevating the SrTiO_3_ by 2 mm above the cavity floor. The maser’s resonant frequency was manually adjustable, centred around the X-Z zero-field transition frequency, ~1.45 GHz.

## Data Availability

The data that support the findings of this study are available from the corresponding author upon reasonable request.
